# The Role of Epigenetic Mechanisms in the Development of PM_2.5_-Induced Cognitive Impairment

**DOI:** 10.3390/toxics13020119

**Published:** 2025-02-02

**Authors:** Lishan Jiang, Mingxia Shao, Chao Song, Li Zhou, Wenke Nie, Hang Yu, Siqi Wang, Yongping Liu, Li Yu

**Affiliations:** 1 Neurologic Disorders and Regenerative Repair Laboratory, Shandong Second Medical University, Weifang 261053, China; lisa3233@163.com (L.J.); shaomingxia030908@163.com (M.S.); songchao720502@163.com (C.S.); zl34952023@163.com (L.Z.); 18253779237@163.com (W.N.); yuhang19990518@163.com (H.Y.); 15881508359@163.com (S.W.); 2 School of Basic Medical Sciences, Shandong Second Medical University, Weifang 261053, China

**Keywords:** PM_2.5_, epigenetic, cognitive impairment

## Abstract

PM_2.5_ is fine particulate matter with a diameter of less than 2.5 μm. Recent evidence has shown that exposure to PM_2.5_ markedly elevates the risk of neurodegenerative diseases, neurodevelopmental disorders, and cardiovascular diseases, which may culminate in cognitive impairment. Nevertheless, the precise mechanisms through which PM_2.5_ affects cognitive function are unclear. Recent studies have demonstrated that PM_2.5_-induced epigenetic alterations are associated with the development of cognitive impairment. Epigenetic alterations include modifications to DNA methylation, histone modifications, and non-coding RNAs. The underlying mechanisms of epigenetic alterations are related to inflammation, synaptic dysfunction, cardiovascular factors, and alterations in neuronal structure and function. This review reports the latest findings on the relationship between PM_2.5_-induced epigenetic alterations and the development of cognitive disorders, offering novel insights into the cognitive effects of air pollution.

## 1. Introduction

A recent assessment by the World Health Organization (WHO) indicated that air pollution is associated with millions of deaths annually, representing the most significant environmental threat to human health. Specifically, particulate matter (PM) is a significant contributor to adverse physical and mental health outcomes. PM_2.5_ has a more pronounced adverse impact on humans than other pollutants [1,2]. Exposure to PM_2.5_ causes about 8.9 million premature deaths yearly worldwide, reducing life expectancy by 2.9 years [3].

**Figure 1:**
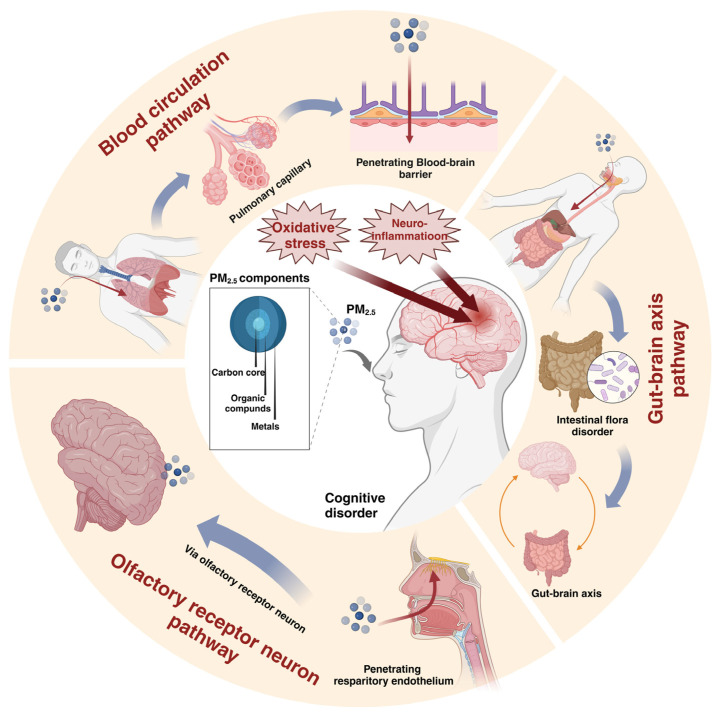
The structure of PM_2.5_ and its pathway into the CNS. PM_2.5_ is composed of a carbon core, organic compounds, heavy metals, and other substances, which can enter the central nervous system through the blood circulation, olfactory neurons, and brain–gut axis. The figure was created with BioRender.com.

Several studies have indicated that exposure to PM_2.5_ is linked to various adverse health outcomes, including airway damage, cardiovascular impairment, heart failure, neurodegenerative diseases, and neurodevelopmental disorders, suggesting that PM_2.5_ mainly affects the respiratory system, cardiovascular system, and nervous system [4]. PM_2.5_ can easily pass through the respiratory epithelium and alveoli due to its small size, large surface area, long residence time, and ability to accumulate heavy metals and toxic organic pollutants in the atmosphere, thus entering blood circulation through the pulmonary capillaries. PM_2.5_ can then enter the central nervous system (CNS) via various primary pathways, thereby impairing brain function. First, PM_2.5_ can enter the CNS by increasing the permeability of the blood–brain barrier (BBB) and decreasing the expression of tight junction proteins. Second, PM_2.5_ can enter the brain through olfactory neurons. Studies have also demonstrated that ultrafine particles (UFPs) migrate along the axons of olfactory nerves and circumvent the BBB, thus penetrating the brain [5]. Furthermore, PM_2.5_ can enter the brain through the gut–brain axis (GBA). Inhaled PM_2.5_ stimulates the hypothalamic release of corticotropin-releasing hormone (CRH) and adrenocorticotropic hormone (ACTH), which in turn synthesize and release cortisol and activate the hypothalamus–pituitary–adrenal (HPA) axis, which is an important component of the GBA. Additionally, PM_2.5_ can enter the gastrointestinal tract, alter the composition and function of gut microbiota, damage the intestinal barrier, cause tryptophan metabolism disorders, and ultimately affect the brain by stimulating the GBA [6]. Recent studies have shown that particulate matter inhaled from the nose and mouth can be transferred to the gastrointestinal tract through multiple routes [7]. A total of 99.95% of particles deposited in the posterior nasal cavity, regardless of size, are transferred to the gastrointestinal tract. Particulate matter deposited in the lungs is transferred from the peripheral lung to the gastrointestinal tract mainly by the mucociliary escalator. A small fraction of particles can still cross the cell membrane from the site of deposition and be distributed throughout the gastrointestinal tract. There is a possibility for a very small fraction of particulate matter to be transported into the pulmonary vasculature and systemic circulation, where it eventually binds to proteins, penetrates the air–blood barrier in the lungs, and reaches the gastrointestinal tract. PM_2.5_ can traverse multiple pathways after its entry, thereby significantly elevating the probability of damage to the nervous system (Figure 1). Therefore, the mechanism by which PM_2.5_ affects the nervous system should be further elucidated to enhance the understanding of the neurotoxicity associated with air pollution.

Studies have demonstrated that PM_2.5_-induced neurological damage is associated with the development of cognitive impairment [8,9]. Notably, cognitive impairment is a prevalent phenomenon in diseases affecting the nervous system, particularly neurodevelopmental disorders (NDDs) and neurodegenerative diseases (NDGDs). Moreover, cardiovascular diseases (CVDs) can impact the nervous system, leading to cognitive impairment. Cognitive impairment is defined as an abnormality in the brain’s higher intellectual processing, and it is related to various functions, such as learning, memory, thinking, and judgment. A properly functioning cerebral cortex is the basis for cognition, indicating that any factor, including inflammation, the dysfunction of neurotransmitter systems, and genetic factors, that alters the structure and function of the cerebral cortex can lead to cognitive impairment [10,11,12,13]. PM_2.5_ causes neurological damage through various mechanisms, such as neuroinflammation, oxidative stress, and mitochondrial dysfunction [14,15]. Nevertheless, the precise mechanism by which PM_2.5_ causes cognitive impairment is unclear.

**Figure 2:**
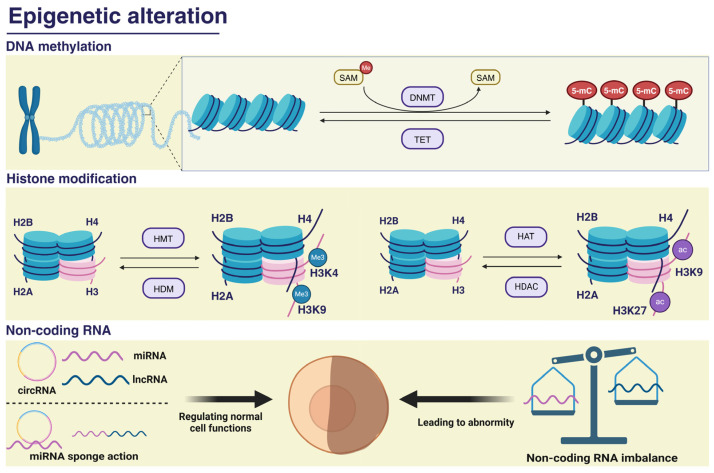
Forms of epigenetic alterations. Epigenetic mechanisms are mainly divided into DNA methylation, histone modification, and non-coding RNA. Among them, DNA methylation refers to the transfer of a methyl group from SAM to the fifth carbon of cytosine residue to form 5-mC catalyzed by DNMT, and the demethylase TET can negatively regulate this process. Histone modification mainly involves histone methylation catalyzed by HMT and histone acetylation catalyzed by HAT. HDM and HDAC negatively regulate these two processes, respectively. Non-coding RNA alterations mainly refer to the change in the NcRNA content through an independent or sponge interaction with NcRNA, which leads to a series of abnormal responses in the body under PM_2.5_ exposure. The figure was created with BioRender.com.

Studies have demonstrated that altered environmental factors can result in epigenetic changes, which may promote cognitive impairment [16,17,18]. Recent studies have demonstrated that PM_2.5_, an environmental factor with pro-inflammatory properties, can induce epigenetic alterations [19,20]. Nevertheless, the precise relationship between cognitive impairment and epigenetic changes remains unclear. Epigenetics is defined as the study of heritable alterations in gene function that do not involve changes in the DNA sequence of the genome. These alterations can affect the phenotype and are mediated by various mechanisms, mainly including DNA methylation, histone modification, and non-coding RNAs (Figure 2). Therefore, PM_2.5_ causes cognitive impairment possibly through epigenetic alterations. This review aimed to provide insights that can inform future research on the harmful effects of air pollution and contribute to the development of new strategies for the prevention and treatment of cognitive dysfunction. Table S1 and Figure S1 shows the the searching string and PRISMA flow chart on article searching, respectively. The criteria of selection and exclusion were determined for the review process according to population, exposure, comparator, and outcomes, or the PECO model. Only research articles containing empirical data were selected (Table S2).

## 2. PM_2.5_-Induced DNA Methylation

**Figure 3:**
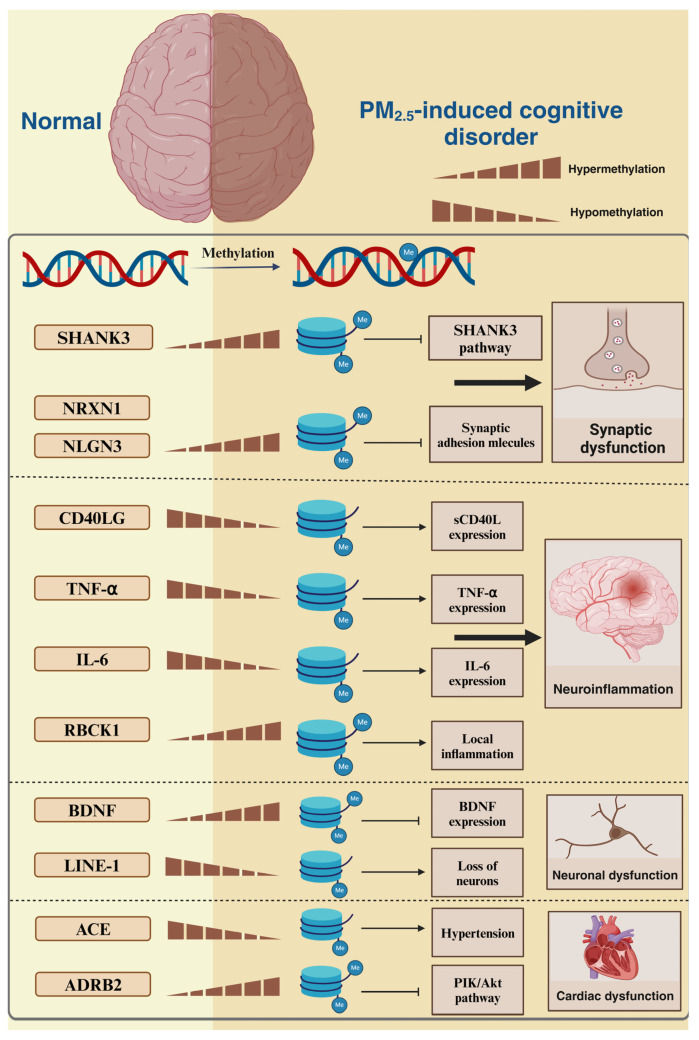
PM_2.5_-induced DNA methylation mechanisms. PM_2.5_ can lead to synaptic dysfunction by down-regulating methylation of SHANK3, NRXN1, and NLGN3 genes. Up-regulation of CD40LG, TNF-α, and IL-6 and down-regulation of RBCK1 gene methylation induced neuroinflammation. Up-regulation of BDNF and LINE-1 gene methylation leads to neuronal dysfunction. Up-regulation of ADRB2 and down-regulation of ACE gene methylation can result in cardiac dysfunction. Figure created with BioRender.com.

DNA methylation is a key epigenetic mechanism within the mammalian genome. DNA methyltransferases (DNMTs), also known as “writers”, catalyze the transfer of a methyl group from S-adenosyl methionine (SAM) to the fifth carbon of cytosine residues, forming 5-methylcytosine (5-mC). DNA methylation can inhibit gene transcription by interfering with the binding of transcription factors to promoters, facilitating the competitive action of transcriptional deterrents, thus altering the structure of chromatin. DNMTs combined with DNA demethylating enzymes (TET1, TET2, and TET3), also known as “erasers”, maintain a complex dynamic equilibrium that regulates gene expression [21]. Alterations in DNA methylation have been linked to a range of adverse effects, including synaptic dysfunction, inflammatory responses, neuronal abnormalities, and alterations in cardiovascular function, which can promote cognitive impairment [22,23,24,25] (Figure 3).

### 2.1. PM_2.5_-Induced DNA Methylation Alterations and Synaptic Dysfunction

Normal synaptic function is a prerequisite for the exercise of brain functions, indicating that minor synaptic dysfunction may lead to neurological dysfunction [23]. Li et al. [26] demonstrated that PM_2.5_-induced DNA methylation changes are associated with reduced synapses. Moderate exposure to PM_2.5_ (224 μg/m^3^) results in the hypermethylation of SH3 and multiple ankyrin repeat domains 3 (SHANK3) in rats during early life stages. This significantly reduces the expression of GKAP and PSD95, key molecules of the SHANK3 signaling pathway, causing autism in rats. SHANK3 is predominantly expressed in the post-synaptic density (PSD) of excitatory glutamatergic neurons, where it forms a molecular bridge by connecting guanylate kinase-associated protein (GKAP) and Homer (a protein constituting the PSD). The PSD forms molecular bridges that are significantly involved in excitatory synapse composition and function [27]. PM_2.5_ may inhibit SHANK3 gene expression by inducing the hypermethylation of SHANK3 in rat brain tissues, which inhibits the SHANK3 signaling pathway and reduces synapses, thus triggering the autism phenotype.

Wei et al. [28] showed that altered DNA methylation is associated with abnormal synaptic homeostasis in human neurons, demonstrating that PM_2.5_ can induce the up-regulation of DNA methylation at the promoters of six genes (BDNF, NRXN1, NLGN3, SHANK3, SLC6A4, and GABRB3). NRXN1 and NLGN3 encode synaptic adhesion molecules that connect pre-synaptic and post-synaptic neurons, respectively, playing a pivotal role in synaptic transmission. The brain can maintain consistent overall synaptic functionality in normal conditions, whereby an increase or decrease in the synaptic strength of a specific neural circuit does not affect brain function. However, a disruption of this equilibrium can result in abnormalities in the neural circuits associated with various neurological disorders, promoting the development of neurological diseases [29,30]. The hypermethylation of NRXN1 and NLGN3 inhibits their expression, thus significantly decreasing the expression of NRXN1 and NLGN3 proteins and other synaptic markers (synapsin1 and PSD-95). This results in an aberrant functional state of synapses and a detrimental impact on synaptic homeostasis. PM_2.5_ can impair the normal function of synapses by affecting the SHANK3 signaling pathway and the methylation of synapse-related genes, leading to the occurrence of cognitive impairment.

### 2.2. PM_2.5_-Induced DNA Methylation Alterations and Inflammation

Several researchers have suggested that inflammatory responses are associated with the development of cognitive impairment [31]. For instance, Chen et al. [32] found that PM_2.5_ may exacerbate the systemic inflammatory response, which can promote cognitive impairment. In a randomized, double-blinded crossover trial of 35 healthy university students in Shanghai, China, short-term exposure to PM_2.5_ resulted in the hypomethylation of 10 candidate genes, including CD40LG. The hypomethylation of CD40LG increases the protein-encoding soluble CD40 ligand (sCD40L), which is involved in the inflammatory and coagulation responses induced by PM_2.5_. Furthermore, recent studies have indicated that sCD40L expression is elevated in the blood of patients with Alzheimer’s disease (AD), indicating that it is a potential biomarker for AD [33]. Therefore, PM_2.5_ may promote the expression of CD40LG, which encodes a protein involved in the inflammatory response, by promoting hypomethylation, thus exacerbating the systemic inflammatory response and increasing the risk of developing cognitive impairment. Additionally, PM_2.5_ can induce the hypomethylation of the CpG site of tumor necrosis factor-α (TNF-α), increasing TNF-α levels within the body. TNF-α is a pivotal pro-inflammatory molecule implicated in the pathogenesis of AD and other neurodegenerative disorders [34,35]. These findings suggest that PM_2.5_-induced DNA methylation may contribute to the development of cognitive abnormalities through systemic inflammatory responses.

Additionally, PM_2.5_-induced DNA methylation alterations can precipitate localized inflammatory responses. Researchers evaluated genome-wide DNA methylation (DNAm) and three AD-related neuropathological markers in prefrontal cortex tissue from 159 donors, discovering that chronic exposure to PM_2.5_ is significantly associated with DNAm differences at the CpG locus of the gene encoding a negative regulator of inflammation, RBCK1 [36]. Furthermore, the CpG locus is positively methylated in nasal cells of children exposed to PM_2.5_, indicating that PM_2.5_ leads to neuroinflammation and nasal inflammation by inducing the hypermethylation of RBCK1, which impedes its inhibitory effect on inflammation [37]. Neuroinflammation is a common feature of various neurological disorders. Nasal inflammation affects cognitive behavior, leading to olfactory bulb atrophy and olfactory sensory neuron degeneration in mice [38,39]. The offspring of female rats exposed to high concentrations of PM_2.5_ exhibit spatial memory deficits and are hypomethylated at the CpG site of the IL-6 promoter, increasing IL-6 levels in the hippocampus [40]. IL-6 overexpression is a hallmark of neuroinflammation, indicating that PM_2.5_ induces the hypomethylation of the IL-6 promoter, resulting in IL-6 overexpression and the promotion of neuroinflammation, thus leading to cognitive impairment in sub-rats.

The aforementioned studies indicate that PM_2.5_ may contribute to cognitive deficits by inducing the hypomethylation of CD40LG, TNF-α, and IL-6 CpG sites and the hypermethylation of RBCK1 CpG sites, thereby triggering systemic or localized inflammatory responses. PM_2.5_ exposure is correlated with the development of a range of neurodegenerative diseases and neurodevelopmental disorders, including AD, Parkinson’s syndrome, and autism, which can manifest as cognitive deficits [41,42]. Numerous studies have demonstrated that neuroinflammation plays a pivotal role in the pathogenesis of neurodegenerative diseases and neurodevelopmental disorders [43,44]. Therefore, further research on DNA methylation alterations may provide novel insights into the underlying mechanisms of PM_2.5_-induced cognitive impairment in neurological disorders.

### 2.3. PM_2.5_-Induced DNA Methylation Alterations and Neuronal Structural and Functional Abnormalities

Neurons are the fundamental structural unit of neural organization and are crucial for the maintenance of optimal cognitive function. Abnormalities in the structure and function of neurons can result in cognitive impairment. A study demonstrated that PM_2.5_-induced alterations in DNA methylation may cause neuronal loss [32]. Furthermore, short-term exposure to PM_2.5_ can result in the hypomethylation of long interspersed nuclear element 1 (LINE-1), a retrotransposon that constitutes approximately 40% of the human genome. These elements can replicate and integrate into new locations throughout the genome, thus disrupting the expression of neighboring genes and the regulatory sequences of exon–intron interactions. The hypomethylation of these transposons enhances their reverse transcriptional translocation, possibly resulting in neuronal loss. In addition, hypomethylation is associated with a range of neurological disorders, including multiple sclerosis, AD, and autism spectrum disorder (ASD) [45,46].

Zhou et al. [47] demonstrated that altered DNA methylation can result in the transgenerational inheritance of reduced BDNF expression, leading to neurodevelopmental disorders in offspring. Maternal exposure to PM_2.5_ during gestation can elevate the expression of Dnmt3a in the hippocampus of first-generation offspring, leading to the hypermethylation of the BDNF gene and the diminished expression of BDNF in the hippocampus of first- to third-generation offspring. Brain-derived neurotrophic factor (BDNF) is widely distributed in the hippocampus and cortex and plays a crucial role in neuronal survival and differentiation and the execution of neuronal functions. Decreased BDNF expression can impair learning, memory, and cognitive abilities [48]. This study also revealed that the first generation of offspring exhibited a heightened susceptibility to alterations in BDNF expression, showing the neurodevelopmental pathological phenotype, which was stably transmitted to the third generation of mice that were not exposed to PM_2.5_, indicating the transgenerational inheritance of PM_2.5_ neurotoxicity across generations. The epigenetic modifications occurring in male and female germline cells can be transmitted across generations under the influence of PM_2.5_ [49]. In view of the effects of maternal exposure on offspring, damage to the placenta should not be ignored. Recent studies have revealed adverse effects of exposure to fine particulate matter on the placenta [50]. Animal studies have shown that PM_2.5_ exposure can cause placental inflammation in rats and mice, suggesting that PM_2.5_ has a toxic effect on the placenta [51,52,53]. A study investigated genome-wide epigenetic changes in human placentas induced by PM_2.5_ exposure and found that placental PM_2.5_-associated DNA methylation changes were enriched in genes involved in immune response and energy metabolism, among which the methylation level of the BID gene increased with PM_2.5_ exposure. BID encodes a death agonist that belongs to the BCL-2 family of cell death regulators and normally forms heterodimers with the agonist BAX or antagonist BCL2 to regulate apoptosis. This study found a 1.396 mm decrease in fetal head circumference for each 1% unit increase in BID methylation. Measurements of head size have been viewed as a marker of fetal brain development, and slow head growth in utero may lead to cognitive dysfunction. Furthermore, BID is susceptible to environmental influences and mediates the release of cytochrome C from cells, causing mitochondrial damage, which may further aggravate placental inflammation [54].

Studies have demonstrated that PM_2.5_ can influence the methylation of genes associated with neuronal activity, resulting in alterations to neuronal structure or function, thus promoting cognitive dysfunction. The transgenerational inheritance of neurodevelopmental disorders indicates that PM_2.5_ neurotoxicity is heritable, thereby providing a novel research avenue for investigating the transmission of PM_2.5_ neurotoxicity between mother and offspring.

### 2.4. PM_2.5_-Induced DNA Methylation Alterations and Cardiovascular Factors

Research has shown that cardiovascular disease is associated with physiologic brain activity, indicating that abnormalities in the cardiovascular system may exacerbate pre-existing neurological disorders or cause new brain damage, which can lead to cognitive dysfunction [55]. In this study, we mainly focused on the impacts of hypertension and cardiac dysfunction (caused by altered DNA methylation-related) on cognitive impairment.

Numerous studies have demonstrated that hypertension can significantly alter the structure and function of cerebral blood vessels. These alterations can lead to ischemia of brain tissues or the production of cognitive deficits (long term) by enlarging the extent of cerebral blood vessels that compress normal brain tissues [56]. Wang et al. [57] showed that hypertension may be induced through altered DNA methylation. Slight exposure to PM_2.5_ is markedly linked to the hypomethylation of the gene encoding the angiotensin-converting enzyme protein (ACE). In addition, the hypermethylation of ACE increases the ACE protein content, promoting vasoconstriction through the renin-angiotensin system, leading to an elevation in blood pressure and an increased risk of cognitive impairment.

Clinical studies have shown that about one-third of patients who develop cognitive impairment also have cardiovascular disease. This may be attributed to the shared risk factors for cognitive impairment and cardiovascular disease. In addition, cardiac dysfunction can directly impact the brain [58]. Cardiac dysfunction caused by cardiomyocyte apoptosis may have a negative impact on an individual’s cognitive status due to inadequate perfusion caused by low cardiac output or inflammatory responses and oxidative stress in the brain [59]. Yang et al. [60] demonstrated that PM_2.5_-induced DNA methylation alterations can promote cardiomyocyte apoptosis, leading to cardiac dysfunction. Furthermore, PM_2.5_ exposure reduces the Bcl-2/BAX ratio and increases apoptotic factor p53 expression in AC16 human cardiomyocytes, thereby promoting apoptosis in AC16. Furthermore, PM_2.5_ may cause cardiac dysfunction in mice. Subsequent studies have demonstrated that PM_2.5_ exposure can result in the hypermethylation of ADRB2, the gene encoding adrenergic receptors (β2 adrenergic receptors, β2ARs), leading to a reduction in the β2AR content and the inhibition of the PI3K/Akt pathway. This may result in a reduction in Bcl-2 expression, which is regulated by the PI3K/Akt pathway, and an attenuation of its inhibitory effect on BAX expression, thus decreasing the Bcl-2/BAX ratio, which leads to apoptosis of AC16 cells [61]. These findings indicate that PM_2.5_-induced DNA hypermethylation promotes cardiomyocyte apoptosis, which leads to cardiac dysfunction.

Numerous studies have shown that air pollution is a significant risk factor for both the incidence and mortality associated with cardiovascular disease. Notably, the presence of cardiovascular disease may significantly indicate air pollution-induced dementia [62]. The aforementioned studies showed that PM_2.5_ can induce cardiovascular disease by modulating DNA methylation levels, thereby jeopardizing cognitive function. This provides novel insights into the potential mechanisms underlying cognitive impairment caused by cardiovascular abnormalities.

In conclusion, PM_2.5_ can modify DNA methylation levels, influence the expression of related genes, and regulate the corresponding proteins. This can result in synaptic dysfunction, inflammation, neuronal dysfunction, and abnormalities in the cardiovascular system, leading to cognitive impairment. Nevertheless, further experimental animal and cellular studies should substantiate the underlying mechanisms by which PM_2.5_-induced DNA methylation alterations contribute to cognitive impairment in other methylation sites.

## 3. PM_2.5_ Alters Histone Modifications and Induces Cognitive Impairment

Histone modification is a key epigenetic regulatory mechanism that influences numerous pathophysiological processes within the human body. Histones can be modified in various ways, including through histone methylation, histone acetylation, and histone ubiquitination. In this study, we emphasized the alterations in histone methylation and acetylation induced by PM_2.5_ and the potential association between histone modification and cognitive impairment.

### 3.1. PM_2.5_-Induced Histone Methylation

Histone methylation occurs at the N-terminal lysine (K) or arginine (R) residues of H3 and H4 histones. The degree of methylation at different sites and the presence of methylation elicit disparate effects, which may be associated with the activation, extension, or repression of gene expression. The methylation process is facilitated by histone methyltransferases (HMTs) and histone demethylases (HDMs). Lysine residues of histones can be mono-, di-, and trimethylated (me1, me2, and me3, respectively), serving as active or repressive markers for gene expression. Trimethylated H3 lysine 4 (H3K4me3) and trimethylated H3 lysine 9 (H3K9me3) affect the activation and repression of gene transcription [63,64]. The potential role of PM_2.5_-induced histone methylation alterations in cognitive impairment is highlighted in the next section, focusing on the underlying inflammatory and hypertensive mechanisms.

#### 3.1.1. PM_2.5_-Induced Histone Methylation and Inflammation

Altered histone methylation is associated with the development of inflammation. Vrijens et al. [65] observed that exposure to PM_2.5_ during pregnancy results in elevated levels of H3K4me3 and total histone H3 in the mother’s cord blood. A study demonstrated that extracellular histones can bind phospholipids, thereby disrupting cell membranes, resulting in persistent calcium influx. Elevated intracellular calcium levels may result in cellular damage and the release of cellular contents, including inflammatory cytokines in leukocytes [66]. Therefore, PM_2.5_-induced high levels of H3K4me3 in umbilical cord blood can cause peripheral inflammation in the fetus, leading to increased blood–brain barrier permeability, the enhanced migration of activated macrophages, and passive diffusion of pathogens and other foreign bodies into the CNS, thereby further exacerbating the existing neuroinflammation. Neuroinflammation is a pivotal mechanism in numerous neurodegenerative disorders and is linked to cognitive decline [43].

#### 3.1.2. PM_2.5_-Induced Histone Methylation and Hypertension

A recent study indicated that altered histone methylation is associated with an elevated pulse pressure. A study examined the effects of chronic exposure to particulate matter from transportation sources on truck drivers and indicated that PM_2.5_ can increase H3K27me3 expression in peripheral blood leukocytes. In addition, chronic exposure to particulate matter was positively correlated with pulse pressure in truckers [67]. These findings indicate that PM_2.5_ may contribute to the development of hypertension by increasing H3K27me3 in peripheral blood leukocytes. Nevertheless, the precise mechanism by which increased histone modifications promote hypertension is unclear. Hypertension can induce cognitive impairment by affecting the structural and functional integrity of the cerebral microcirculation [56]. These findings indicate that PM_2.5_ induces hypertension by increasing peripheral blood leukocyte H3K27me3, which leads to the development of cognitive impairment. Notably, the prevalence of hypertension is high in populations at risk of developing cognitive impairment [68]. Further research into the epigenetic molecular mechanisms by which PM_2.5_ causes hypertension may provide a new direction for subsequent research into the alleviation of PM_2.5_ neurotoxicity.

### 3.2. PM_2.5_-Induced Histone Acetylation

Histone acetylation is a post-translational modification that occurs predominantly at specific lysine residues situated within the N-terminal basic amino acid concentration region of core histones. This process is regulated by histone acetyltransferases (HATs) and histone deacetylases (HDACs). HATs can activate gene transcription through histone acetylation, which reduces the positive charge of lysine residues, thereby inhibiting the binding of histone tails to negatively charged DNA and weakening the binding of the underlying DNA. The resulting exposure of the underlying DNA and weakening of the electrostatic attraction between DNA and histones are the underlying mechanisms of this process. In contrast, HDACs deacetylate histones inhibit gene transcription. Histone 3 lysine 27 acetylation (H3K27ac) and histone 3 lysine 9 acetylation (H3K9ac) are associated with AD-associated pathways that can lead to cognitive impairment [64,69]. The role of PM_2.5_-induced histone acetylation changes in cognitive impairment is highlighted in the next section, and two underlying mechanisms are delineated: amyloid toxicity and alterations in cardiovascular function.

#### 3.2.1. PM_2.5_-Induced Histone Acetylation and Amyloid Toxicity

Amyloid accumulation in the brain is a key characteristic of AD and is a primary cause of dementia [70]. PM_2.5_-induced elevations in histone acetylation can exacerbate amyloid toxicity. Ding et al. [71] observed that PM_2.5_ can increase H3K9ac levels in peripheral blood mononuclear cells (PBMCs) by increasing reactive oxygen species (ROS) in rats. The elevated acetylation of PBMCs suggests that the overall acetylation level of the body is increased. A study revealed that H3K27ac and H3K9ac levels are elevated in the brain tissue of patients with AD. Furthermore, increased H3K27ac and H3K9ac levels exacerbate the toxicity of amyloid beta-protein 42 (A-β42) in drosophila [69]. These findings indicate that PM_2.5_ increases H3K9ac levels in the body, thereby increasing the risk of cognitive impairment in the brain by exacerbating amyloid toxicity. This provides epigenetic evidence for the association between air pollution and neurodegeneration.

#### 3.2.2. PM_2.5_-Induced Histone Acetylation and Cardiovascular Factors

Pathologies affecting the cardiovascular system, such as hypertension and cardiac dysfunction, are associated with the onset of cognitive impairment. A study demonstrated that PM_2.5_-induced alterations in histone H3K27 acetylation may trigger the development of hypertension. PM_2.5_ may induce elevated levels of H3K27ac, a specific gene involved in platelet activation, blood coagulation, and hemostasis. This promotes the expression of these genes and increases erythrocyte coagulation, leading to hypertension, thus increasing the risk of cognitive impairment [72]. Furthermore, altered histone acetylation may result in cardiac dysfunction [73]. Studies have demonstrated that the exposure of pregnant mice to PM_2.5_ reduces the levels of H3K9 acetylation bound to the promoter region of the GATA4 gene in the hearts of the offspring, leading to a decline in the expression of the GATA4 gene. GATA4 is a pro-hypertrophic transcription factor, which is regarded as a pivotal mediator of cardiac gene transcription. GATA4 down-regulation may result in cardiac damage and subsequent cardiac dysfunction [74]. The findings indicate that PM_2.5_-induced alterations in histone acetylation may promote the development of cognitive impairment by causing hypertension and cardiac dysfunction.

**Figure 4:**
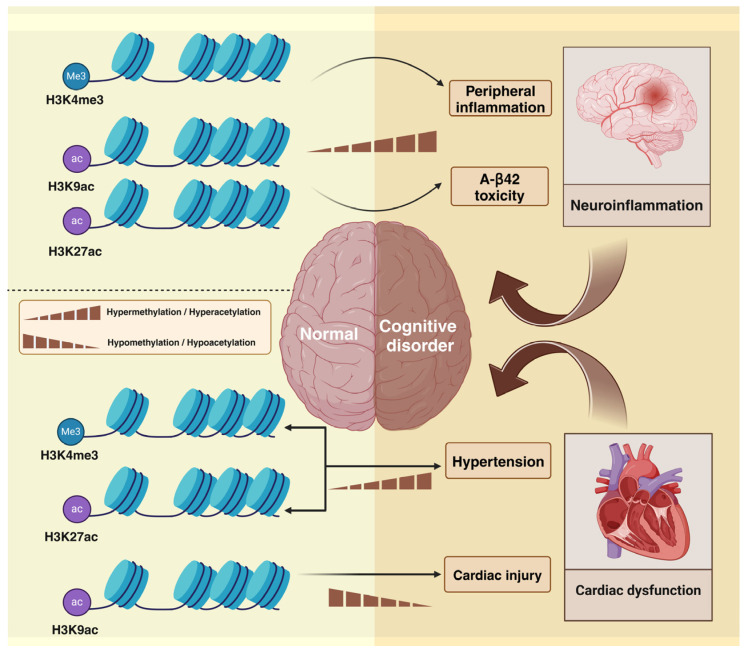
PM_2.5_-induced histone modification mechanisms. In this study, we mainly focus on histone methylation and histone acetylation. PM_2.5_ caused neuroinflammation by increasing the contents of H3K4me3, H3K9ac, and H3K27ac. Increased H3K4me3 and H3K27ac contents and a decreased H3K9ac content can result in cardiac dysfunction. The figure was created with BioRender.com.

In conclusion, PM_2.5_ can alter histone methylation and acetylation levels, thereby increasing the risk of cognitive impairment through inflammation, cardiovascular system abnormalities, and amyloid toxicity (Figure 4). Nevertheless, most recent studies have conducted extensive proteomic analyses. Therefore, further animal experiments and epidemiological studies should fully elucidate the association between PM_2.5_-induced histone modification alterations and cognitive impairment.

## 4. PM_2.5_-Associated Non-Coding RNA and Cognitive Impairment

Non-coding RNAs (ncRNAs) are prevalent in a wide variety of organisms. ncRNAs are thought to lack protein-coding functions. LncRNA, miRNA, and CircRNA are mainly involved in post-transcriptional regulation. Several studies have found that miRNA and LncRNA levels change in patients with cognitive disorders, such as AD and Parkinson’s disease (PD) [75,76].

In addition, studies have shown that ncRNAs play a great role in the development of neurocognitive disorders [77,78,79,80,81]. Moreover, PM_2.5_ can cause an abnormal regulation of ncRNAs, triggering different pathological mechanisms, leading to the manifestation of cognitive deficits [82,83]. These findings describe the involvement of PM_2.5_-induced ncRNA changes in cognitive deficits through inflammation, synaptic dysfunction, oxidative stress and Aβ deposition, and tau protein hyperphosphorylation.

### 4.1. PM_2.5_-Associated Non-Coding RNA Alterations and Inflammation

Recent studies have shown that non-coding RNA changes caused by PM_2.5_ exposure increase inflammation in the brain through two main pathways. The first pathway involves the gradual progression of the inflammatory response from the local periphery to organs throughout the body, including brain tissue, through certain cytokines and inflammatory mediators [84]. Local inflammation activates macrophages and monocytes, which produce a series of cytokines (TNF-α and IL-6) that act as molecular mediators to propagate the response throughout the body. The second pathway involves PM_2.5_ exposure stimulating some parts of the brain’s structures, which directly induces an inflammatory response in the brain, leading to associated cognitive impairment disorders. For the first pathway, studies have found that peripheral inflammation increases the susceptibility of midbrain dopaminergic neurons to noxious stimuli. Cytokines, such as IL-1b, TNF-α, and IL-6, in circulation may play a relevant role in the intensification of central (brain) inflammation. In addition, peripheral inflammation plays a key role in disrupting the integrity of the blood–brain barrier, facilitating the entry of peripheral cytokines into the CNS, triggering neuroinflammation [85,86]. The inflammatory factor TNF-α, which plays a role in this process, can initiate a peripheral cytokine release cascade, leading to cognitive impairment. IL-1β can disrupt the blood–brain barrier, facilitating the entry of inflammatory factors into the brain to damage neurons. Inflammation-related pathways, such as increased nuclear factor κB (NF-κB), can promote the production of the above inflammatory factors, thus promoting cognitive impairment. Since PM_2.5_ is inhaled, peripheral inflammation is mainly caused by the diffusion of inflammation in the lungs, which further causes cognitive impairment. Liu et al. confirmed that PM_2.5_ exposure induces differential miRNA expressions in pulmonary epithelial cells via in vivo and in vitro assays. Some differentially expressed miRNAs were transferred into alveolar macrophages to regulate the transcription of inflammatory genes and activate inflammatory pathways, resulting in a local inflammatory micro-environment. Another part of miRNA transferred into adjacent alveolar epithelial cells regulated the expression of target genes in cell damage signaling pathways, accelerated local lung tissue injury, and increased local vascular permeability by destroying the air–blood barrier, aggravating lung inflammation. The inflammatory factors produced in this process can enter the blood and mediate the damage of the whole lung and other tissues and organs [87]. Jardim et al. [88] demonstrated that PM_2.5_ induces alterations in miRNA expression, up-regulating specific miRNAs while down-regulating others, which in turn activates inflammatory pathways. This finding suggests that miRNA-mediated responses to external damage involve a complex regulatory network of genes, which must remain balanced to coordinate cellular responses effectively. In this review, we focus on the primary pathway by which these changes contribute to neuroinflammation.

PM_2.5_-induced changes in miRNAs can lead to alterations in inflammation-related pathways, such as NF-κB, causing bronchial inflammation and myocardial inflammation. Aberrant regulation of NF-κB is associated with inflammation, synaptic plasticity, and memory processes. Li et al. [83] suggested that PM_2.5_-induced inflammation in mice is mainly related to alterations in NF-κB and mitogen-activated protein kinase (MAPK) pathways. In addition, studies have shown that PM_2.5_ contributes to inflammation in human bronchial epithelial cells (BEAS-2B) and mice with chronic obstructive pulmonary disease (COPD) by significantly down-regulating miR-149-5p and up-regulating the expression levels of genes and proteins related to NF-κB and MAPK. Feng et al. [89] demonstrated that PM_2.5_ exposure significantly down-regulates miR-205 in cardiomyocytes (AC16), which activates the IRAK2/TRAF6/NF-kB signaling pathway, causing cardiomyocyte apoptosis and myocardial inflammation. These peripheral inflammations lead to the progression of the inflammatory response to the whole body, especially the brain, through inflammatory factors and cellular mediators, as well as by altering the NF-κB pathway, which leads to cognitive dysfunction.

PM_2.5_ exposure causes lncRNA alterations, resulting in the activation of inflammatory pathways and cellular oxidative damage. Li et al. [90] reported that PM_2.5_ up-regulated circ104250 and lncRNA uc001.dgp.1 in human bronchial epithelial cells (BEAS-2B), which functioned as “drivers” to down-regulate the expression of miR-3607-5p and up-regulate the levels of interleukin 1 receptor (IL1R1) gene and protein. This leads to the activation of the NF-κB signaling pathway and inflammatory responses. Using human bronchial epithelial cells, Roundtree demonstrated that PM_2.5_ treatment promoted the expression of lncRNA linc01515 by enhancing 6-methyladenosine (m6a) modification, which in turn enhanced oxidative damage in airway epithelial cells via the nuclear factor erythrocyte 2 related factor 2 (Nrf2) [91]. Exposure to high concentrations of PM_2.5_ exacerbated pulmonary oxidative stress in mice with left heart failure and increased IL-10 mRNA in mice lungs [92]. In vitro and in vivo experiments have demonstrated that PM_2.5_ enhances macrophage activation and promotes lung inflammation by down-regulating the expression of the non-coding RNA lncgm16410 [93]. It also up-regulates the expression of lncRNA AABR07005593.1, which activates the NF-κB signaling pathway, thereby promoting the expression of IL-6 and lung inflammation [94].

Alterations in miRNAs regulate macrophage polarization to alter inflammatory responses. Xie et al. [95] found that PM_2.5_ altered miRNA expression in vivo and enhanced the enrichment of miR-217-5p and the JAK-STAT signaling pathway. The phosphorylation of the signal transducer and activator of transcription 1 (STAT1) in the cytoplasm forms a dimer, which translocates to the nucleus to promote the expression of certain target genes, stimulating macrophage M1-type polarization and amplifying inflammatory responses. In contrast, the miR-217-5p mimic suppressed ROS levels in the lung tissues of PM_2.5_-treated mice, indicating that it might become a future therapeutic target for alleviating the inflammatory response triggered by PM_2.5_ exposure to some degree [83].

It is interesting to note that people with COPD or asthma have higher risks of dementia and cognitive impairment [96]. PM_2.5_ can contribute to both of them. Firoozi et al. [97] found that direct exposure to PM_2.5_ had a negative impact on lung tissue and exacerbated pulmonary inflammation in COPD model mice. The detailed mechanism is that circBbs9 exacerbates lung inflammation in response to PM_2.5_ exposure and tumor NLRP3 by binding to miR-30e-5p. Furthermore, circBbs9 binding to miR-30e-5p prevented Adar protein from being degraded, activating the NLRP3 inflammation and boosting the production of inflammatory cytokines, after exposure to PM_2.5_. Huang et al. [98] found that acute PM_2.5_ exposure can activate the Notch1-GATA3 pathway in a model of asthma bronchial epithelial cells, which might be involved in PM_2.5_-induced asthma exacerbation. miR-139-5p has a potential protective role in inhibiting PM_2.5_-induced asthma airway inflammation by targeting Notch1. Wang et al. [99] confirmed that PM_2.5_ inhibits SOD1 expression by up-regulating microRNA-206 and promotes ROS accumulation and disease progression in asthmatic mice. miR-206 can target the 3′-UTR of SOD1 to inhibit SOD1 expression, which leads to the increase in ROS level and aggravates the pulmonary inflammatory response and asthma symptoms.

In summary, PM_2.5_ exposure can trigger local inflammation by inducing non-coding RNA changes, altering the inflammatory factors in blood circulation. The entry of inflammatory factors in the brain may cause neuroinflammation and cognitive disorders often detected in AD and PD.

### 4.2. PM_2.5_-Associated Non-Coding RNA Alterations and Synaptic Dysfunction

Although neurodegenerative diseases are often diagnosed many years after their onset, synaptic dysfunction occurs before symptom onset and is therefore used as a marker of cognitive impairment in cases where cognitive symptoms are mild or undetectable [100]. It has been reported that ncRNA changes induced by PM_2.5_ exposure alter the synaptic function. The following section focuses on the mechanisms by which ncRNA modulates the expression of neurotrophic factors and neurotoxic peptides to cause synaptic dysfunction.

Alterations in miRNAs have been reported to alter the secretion of neurotrophic factors. Liu et al. [101] found that exposure to PM_2.5_ during neural development decreased the viability of immature and mature hippocampal neurons, enhanced neuronal apoptosis, and disrupted the synaptic ultrastructure and the expression of synapse-associated proteins. Numerous epidemiological studies have demonstrated that PM_2.5_ may exert neurotoxic effects on the brain to induce central nervous system damage and neurodevelopmental disorders, such as autism spectrum disorders and neuropsychiatric disorders such as AD and PD [15,102,103,104]. The protein kinase A (PKA)/cyclic adenosine monophosphate-responsive element binding protein (CREB)/BDNF signaling pathway may mediate PM_2.5_-mediated neurodevelopmental toxicity. BDNF is one of the most widely distributed and studied neurotrophic factors in the mammalian brain, which regulates the normal growth, development, and plasticity of glutamatergic and γ-aminobutyric acidergic synapses and modulates 5-hydroxytryptaminergic and dopaminergic neurotransmission by affecting neuronal differentiation. BDNF may alter functional and structural plasticity in the CNS, modulating dendritic spines, at least in the hippocampus, thereby altering adult neurogenesis. Several studies have documented a negative feedback loop of regulation between BDNF and numerous miRNAs. Seven BDNF-related miRNAs (miR-15a, miR-206, miR-155-5p, miR-16, miR-103-3p, miR-330-3p, and Let-7a-3p) were identified through the high-throughput sequencing of different brain regions or neurological diseases. Following PM_2.5_ exposure, these miRNAs exhibit differential changes in expression, thereby regulating BDNF to promote cognitive disorders and diseases. The regulatory role of the PKA/CREB/BDNF pathway in PM_2.5_-mediated neurodevelopment has been observed in vitro, but further in vivo experimental studies are warranted [101].

Several miRNAs modulate the expression of neurotoxic peptides to cause synaptic dysfunction. aβ is a neurotoxic peptide formed by the excision of the β-site cleavage enzyme 1 (BACE1) and γ-secretase complex of the β-amyloid precursor protein from amyloid precursor protein (APP), which serves as a marker of early cognitive deficits and is considered an important target for regulating cognitive deficits [93]. Chopra et al. [105] showed that miR-298 was an inhibitor of APP and BACE1, which decrease the production of Aβ to protect synaptic function. This indicates that miR-298 may be a potential therapeutic target. Another animal study showed that the expression level of miR-574-5p in the hippocampus was regulated by NF-κB p65, and the down-regulation of miR-574-5p expression in the hippocampus after PM_2.5_ exposure up-regulated the level of BACE1, inducing neurotoxicity and disrupting the integrity of synaptic function [106].

In summary, the data reviewed here indicate that non-coding RNAs altered by PM_2.5_ may influence synaptic function and cognitive deficits by influencing neurotrophic factors and neurotoxic peptides, suggesting that they might be potential targets for the treatment of PM_2.5_-associated cognitive deficits in the future.

### 4.3. PM_2.5_-Associated Non-Coding RNA Alterations and Aβ Deposition and Tau Protein Hyperphosphorylation

Studies have shown that AD belongs to the group of neurocognitive disorders, which are characterized by high Aβ deposition and the hyperphosphorylation of tau proteins. Thus, the levels of amyloid β peptide (Aβ42) and phosphorylated tau proteins (p-tau) in the brain are considered important diagnostic markers of early AD [107,108].

Alterations in non-coding RNA modulate Aβ deposition. Notably, Aβ can increase the generation of free radicals within neurons and induce oxidative damage and neuronal death by increasing the generation of pro-inflammatory cytokines and enzymes; either way, it may cause cognitive impairment-related disorders [109]. Furthermore, based on the β-amyloid hypothesis of AD, brain Aβ accumulation triggers a cascade of events leading to cognitive deficits and dementia [110]. Hou et al. [111] found that PM_2.5_ exposure resulted in the up-regulation of 10 differentially expressed miRNAs, among which miR-340 was postulated to decrease Aβ accumulation by targeting BACE1, thereby alleviating cognitive dysfunction in patients with AD [112,113,114]. As mentioned earlier, Aβ is cut by the “scissors” of BACE1, and therefore, the inhibition of the activity of the “scissors” decreases the amount of cut Aβ.

In addition, non-coding RNAs affect the levels of p-tau protein, which, when phosphorylated, can cause dysfunction and reduce the viability of neurons [115]. It has been postulated to be correlated with the occurrence of neurodegeneration and cognitive decline [116].

The findings from the study by Monika Grigorova et al. show a synergistic relationship between Aβ and tau proteins in memory and spatial cognitive changes, which is in line with the fact that increased Aβ production in AD increases the phosphorylation level of tau protein [117]. Growing evidence indicates that the neurotoxicity of Aβ triggers the hyperphosphorylation of tau proteins via activating the signaling cascades, which are normally associated with neuronal cell cycle activation and the hyperactivation of specific transcription factors such as c-Myb and PAX6 [118]. Exposure to PM_2.5_ increased the expression of total tau protein (T-tau) and p-tau in the olfactory bulb in animal models. Moreover, another study demonstrated that prenatal exposure to PM_2.5_ enhanced the expression of p-tau, causing pathological damage to the cerebral cortex in the offspring.

Taken together, these findings indicate that PM_2.5_ exposure-induced alterations in non-coding RNAs contribute to the typical pathological features of various neurocognitive disorders. Recent studies have also identified some therapeutic targets for patients with AD; however, the exact pathways affected downstream of the Aβ-induced cell cycle imbalance are still unknown and deserve further investigation.

### 4.4. PM_2.5_-Associated Non-Coding RNA Alterations and Oxidative Stress

Accumulating evidence suggests that PM_2.5_ exposure may induce systemic oxidative stress in human or animal cells, and oxidative stress is considered to be one of the key mechanisms leading to cognitive deficits in neurodegenerative diseases such as AD [119]. Therefore, PM_2.5_-induced oxidative stress is currently considered to be an important mechanism of PM_2.5_-mediated neurotoxicity. This is because the several organic chemicals encapsulated on the surface of PM_2.5_ can be metabolically activated to REMs to generate or increase intracellular ROS [120]. Although ROS have beneficial physiological roles, they may have toxic effects on the body at high concentrations. The excessive production of oxidative stressors can make them gradually accumulate throughout a person’s life, damaging various parts of the body, especially the central nervous system. Secondly, oxidative stressors may arise from the PM_2.5_-mediated activation of inflammatory cells, increasing the levels of ROS and reactive nitrogen species (RNS) [121]. As previously mentioned, the inflammatory response triggered by PM_2.5_ can directly induce cognitive impairment, and it also mediates the associated neurotoxicity through the production of ROS. Third, PM_2.5_ exposure can compromise the body’s antioxidant defense system, diminishing the cellular antioxidant capacity. Nrf2, a key sensor of oxidative and electrophilic stress, is recognized as a critical regulator of cellular responses to environmental stressors. The activation of the Nrf2 signaling pathway stimulates the expression of numerous antioxidant and detoxifying enzyme genes, enhancing the cell’s ability to combat oxidative damage [91]. Kumari et al. [122] reported that Nrf2 modulated the expression of several miRNAs, such as miR-21-5p, miR-126-3p, and miR-100-5p, which in turn affected the expression of various antioxidant enzyme genes. A study by Wang et al. [123] showed that PM_2.5_ treatment up-regulated the expression of linc01515 by enhancing its m6a modification, which in turn exacerbated Nrf2-induced oxidative damage in airway epithelial cells. Fourth, PM_2.5_ exposure influences the levels of antioxidant enzymes, including superoxide dismutase (SOD), catalase (CAT), and glutathione peroxidase (GSH-Px), thereby reducing their activities. In female asthmatic mice, intratracheal drip of PM_2.5_ promoted ROS accumulation and inflammation by up-regulating the expression of miRNA-206 and inhibiting the translation of SOD1 [99]. In addition, miR-217-5p mimics reduced ROS levels in the lung tissues of PM_2.5_-treated mice, suggesting that they may be future therapeutic targets for alleviating ROS caused by PM_2.5_ exposure. Excess ROS may activate several signaling pathways, such as NF-κB, causing a range of adverse effects on cells, but interestingly, these adverse effects can be inhibited or attenuated by antioxidants [124].

In conclusion, PM_2.5_ can alter the levels of non-coding RNAs and increase the risk of cognitive impairment by inducing neuroinflammation, synaptic dysfunction, oxidative stress, Aβ deposition, and the hyperphosphorylation of tau protein (Table 1). However, the mechanisms underlying the interactions between various non-coding RNAs and various pathways remain elusive. Further cellular and animal experiments are needed to determine the relationship between PM_2.5_-induced changes in non-coding RNAs and cognitive impairment.

## 5. Role of Harmful Components of PM_2.5_ in Cognitive Impairment

**Figure 5:**
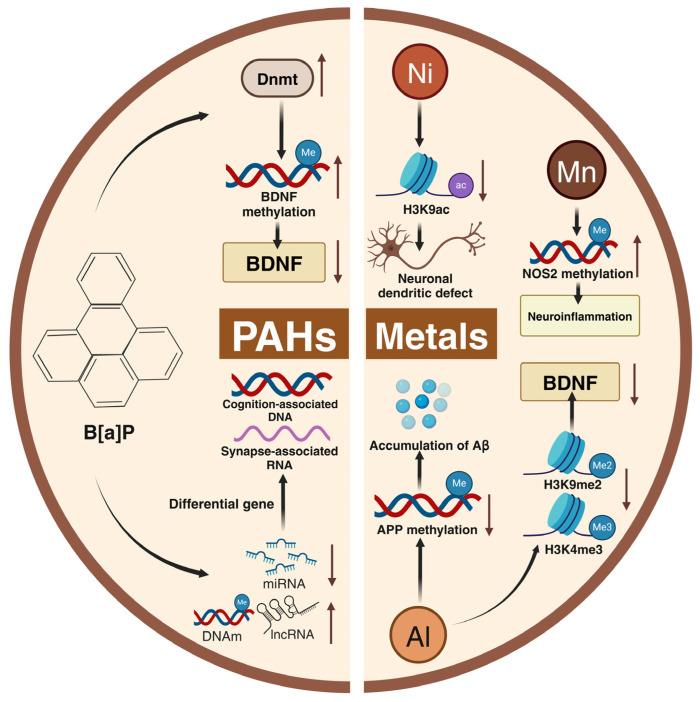
Metals and PAHs induced epigenetic alterations. Metals and PAHs are the main toxic components of PM_2.5_. In PAHs, B[a]P can up-regulate the expression of Dnmt and BDNF gene methylation, leading to the reduction in the BDNF content. It is also able to up-regulate DNA methylation, increase the lncRNA content, and decrease the miRNA content, leading to the differential expression of synapse-related RNA and cognitive-related DNA. Among the metal elements, Al down-regulates the methylation of the APP gene, leading to Aβ deposition. It can also decrease H3K9me2 and H3K4me3, resulting in the reduction in the BDNF content. Ni reduces the H3K9ac content, leading to neuronal dendritic defects. Mn up-regulates the methylation of the NOS2 gene, causing neuroinflammation. The figure was created with BioRender.com.

PM_2.5_ particles contain a variety of toxic substances that, once inhaled, can inflict significant damage on the nervous system. Researchers are increasingly examining the harmful components of these fine particles and their potential neurotoxic effects. This paper mainly discusses the epigenetic mechanism of cognitive impairment following exposure to heavy metals and polycyclic aromatic hydrocarbons (PAHs), which are the main components of PM_2.5_ (Figure 5).

### 5.1. Heavy Metals

Short-term exposure to PM_2.5_ was reported to cause neurological damage, owing to its elements such as Al, Ni, and Mn [125]. It has also been reported that AlCl3 treatment down-regulated DNA methylation-related genes in mice, reduced the hypomethylation of the APP, up-regulated its expression, and promoted amyloid accumulation, leading to cognitive impairment [126]. Studies investigating aluminum workers as subjects revealed that environmental exposure damaged cognitive function by reducing histone H3K4me3 and H3K9me2 levels, which decreased the brain’s cognition-related neurotrophic factors BDNF and EGR1 [127]. Zhou et al. [128] demonstrated that Ni can cause neuronal dendritic defects by down-regulating the levels of H3K9ac in mice, which in turn causes cognitive impairment. Mn decreased the DNA methylation of carbon monoxide synthase 2 (NOS2) exons and promoted its expression to induce neuroinflammation and promote cognitive impairment [129].

### 5.2. PAHs

The toxic effects of PAHs on the nervous system have long been recognized, with benzo[a]pyrene (B[a]P) being the most studied [130]. Studies have demonstrated that B[a]P-treated mice exhibited increased levels of Dnmt in the cerebral cortex, causing hypermethylation of the BDNF gene and decreasing BDNF in the brain, resulting in cognitive deficits in mice [131]. Wang et al. [132] found that exposure to B[a]P triggered a global increase in DNA methylation and lncRNA levels while reducing mRNA and miRNA expression in the mouse hippocampus. Differentially methylated genes were linked to learning and memory impairments, whereas altered RNA expression was found to impact synaptic function.

In conclusion, heavy metal elements and organic components in PM_2.5_ can indirectly trigger cognitive impairment by causing inflammation and promoting amyloid toxicity and neuronal deficits via modulating epigenetic alterations and directly by altering the expression of cognition-related genes. However, different components of the chemical components of PM_2.5_ have distinct effects on the body. Therefore, further research is needed to explore the neurotoxicity associated with various components of PM_2.5_.

## 6. Conclusions

The World Health Organization (WHO) has set higher requirements for atmospheric PM_2.5_ and ozone concentrations: the daily guideline value for PM_2.5_ is 15 µg/m^3^, and the annual average value is 5 µg/m^3^. Air pollution, often referred to as the “invisible killer,” has long posed a significant threat to public health. Although the air quality has improved, the harmful effects of air pollutants remain a challenge.

Epigenetics is considered a potential mechanism governing environment–gene interactions. The effects of PM_2.5_ on epigenetic mechanisms, e.g., DNA methylation, histone modification, and non-coding RNA, are increasingly being studied. Therefore, these studies provide several targets for treating diseases caused by PM_2.5_.

In this review, we provide a comprehensive analysis of epidemiological data and experimental evidence from both in vivo and in vitro studies, with a focus on the epigenetic mechanisms underlying PM_2.5_-induced cognitive impairment. Specifically, we examine how PM_2.5_ exposure influences DNA methylation, histone modifications, and non-coding RNA expression, highlighting the links between harmful PM_2.5_ components and these epigenetic alterations. Exposure to PM_2.5_ alter the levels of lnCRNAs and cirCRNAs to promote cognitive impairment through mechanisms including inflammation and oxidative stress, which suggests the possibility of “the same amount and different quality” principle at play.

Recent research has revealed epigenetic changes in the body as a whole, with only a few animal and cell experiments focusing on specific epigenetic mechanisms. Given that the effects of PM_2.5_ are mediated by multiple mechanisms, further in-depth research is needed to improve our understanding of these mechanisms in cognitive impairment.

## Figures and Tables

**Table 1:** The alteration in non-coding RNAs under PM_2.5_ exposure.

Name	Regulate	Output	Ref.
miR-149-5p	↓	miR-149-5p plays a crucial role in PM_2.5_-induced inflammation.	[76]
miR-217-5p	↓	miR-217-5p mimics can reduce the level of ROS in lung tissues of mice treated with PM_2.5._	[87]
miR-298	↓	It can inhibit the expression of neurotoxic peptides.	[92]
miR-574-5p	↓	PM_2.5_-induced down-regulation of mir-574-5p causes neurotoxicity and further disrupts the integrity of synaptic function.	[93]
miR-340	↑	PM_2.5_-induced up-regulation of miR-340 can reduce the accumulation of amyloid-β and reduce cell apoptosis by targeting BACE1.	[99]
miR-135a	↑	PM_2.5_-induced up-regulation of miR-135a can inhibit the expression and activity of BACE-1.	[101]
miR-21-5p miR-126-3p miR-100-5p	\ \ \	They are the miRNAs most regulated by Nrf2.	[109]
miR-206	↑	PM_2.5_-induced up-regulation of miRNA-206 inhibits the translation of superoxide dismutase 1 (SOD1), leading to ROS accumulation and inflammation.	[111]
miR-205	↓	PM_2.5_-induced down-regulation of miR-205 activates the IRAK2/TRAF6/NF-kB signaling pathway, causing cardiomyocyte apoptosis and myocardial inflammation.	[81]
uc001.dgp.1	↑	PM_2.5_ will up-regulate lncRNAuc001.dgp.1 to inhibit the expression of miR-3607-5p.	[82]
linc01515	↑	PM_2.5_-induced up-regulated linc01515 regulates NrF2-induced oxidative damage in airway epithelial cells, ultimately leading to inflammation.	[110]
lncgm16410	↓	PM_2.5_-induced down-regulation of lncgm16410 enhances the activation of macrophages and promotes lung inflammation.	[85]
AABR07005593.1	↑	PM_2.5_-induced up-regulation of AABR07005593.1 is involved in the activation of the κ-B signaling pathway, which ultimately promotes the expression of IL-6 and causes inflammation.	[86]
circ104250	↑	PM_2.5_ will up-regulate circ104250 to inhibit the expression of miR-3607-5p.	[82]

## Data Availability

The authors confirm that the data supporting the findings of this study are available within the article.

## Supplementary Materials

The following supporting information can be downloaded at: https://www.mdpi.com/article/10.3390/toxics13020119/s1, Figure S1: The searching string; Table S1: PRISMA flow chart on article searching; Table S2: PECO (population, exposure, comparator, and outcome) model.

## References

[1] Liu C., Chen R., Sera F., Vicedo-Cabrera A.M., Guo Y., Tong S., Coelho M.S.Z.S., Saldiva P.H.N., Lavigne E., Matus P. (2019). Ambient Particulate Air Pollution and Daily Mortality in 652 Cities. N. Engl. J. Med..

[2] Li J., Tang W., Li S., He C., Dai Y., Feng S., Zeng C., Yang T., Meng Q., Meng J. (2023). Ambient PM2.5 and its components associated with 10-year atherosclerotic cardiovascular disease risk in Chinese adults. Ecotoxicol. Environ. Saf..

[3] Feng S., Huang F., Zhang Y., Feng Y., Zhang Y., Cao Y., Wang X. (2023). The pathophysiological and molecular mechanisms of atmospheric PM2.5 affecting cardiovascular health: A review. Ecotoxicol. Environ. Saf..

[4] He B., Xu H.-M., Liu H.-W., Zhang Y.-F. (2023). Unique regulatory roles of ncRNAs changed by PM2.5 in human diseases. Ecotoxicol. Environ. Saf..

[5] Wang J., Ma T., Ma D., Li H., Hua L., He Q., Deng X. (2021). The Impact of Air Pollution on Neurodegenerative Diseases. Ther. Drug Monit..

[6] Li T., Fang J., Tang S., Du H., Zhao L., Wang Y., Deng F., Liu Y., Du Y., Cui L. (2022). PM2.5 exposure associated with microbiota gut-brain axis: Multi-omics mechanistic implications from the BAPE study. Innovation.

[7] Zhao L., Fang J., Tang S., Deng F., Liu X., Shen Y., Liu Y., Kong F., Du Y., Cui L. (2022). PM2.5 and Serum Metabolome and Insulin Resistance, Potential Mediation by the Gut Microbiome: A Population-Based Panel Study of Older Adults in China. Environ. Health Perspect..

[8] Meo S.A., Salih M.A., Al-Hussain F., Alkhalifah J.M., Meo A.S., Akram A. (2024). Akram Environmental pollutants PM2.5, PM10, carbon monoxide (CO), nitrogen dioxide (NO2), sulfur dioxide (SO2), and ozone (O3) impair human cognitive functions. Eur. Rev. Med. Pharmacol. Sci..

[9] Ke L., Zhang Y., Fu Y., Shen X., Zhang Y., Ma X., Di Q. (2022). Short-term PM2.5 exposure and cognitive function: Association and neurophysiological mechanisms. Environ. Int..

[10] Ball H.A., McWhirter L., Ballard C., Bhome R., Blackburn D.J., Edwards M.J., Fleming S.M., Fox N.C., Howard R., Huntley J. (2020). Functional cognitive disorder: Dementia’s blind spot. Brain.

[11] Rundek T., Tolea M., Ariko T., Fagerli E.A., Camargo C.J. (2021). Vascular Cognitive Impairment (VCI). Neurotherapeutics.

[12] Velikonja T., Fett A.-K., Velthorst E. (2019). Patterns of Nonsocial and Social Cognitive Functioning in Adults With Autism Spectrum Disorder. JAMA Psychiatry.

[13] Morozova A., Zorkina Y., Abramova O., Pavlova O., Pavlov K., Soloveva K., Volkova M., Alekseeva P., Andryshchenko A., Kostyuk G. (2022). Neurobiological Highlights of Cognitive Impairment in Psychiatric Disorders. Int. J. Mol. Sci..

[14] Nunez Y., Boehme A.K., Weisskopf M.G., Re D.B., Navas-Acien A., van Donkelaar A., Martin R.V., Kioumourtzoglou M.-A. (2021). Fine Particle Exposure and Clinical Aggravation in Neurodegenerative Diseases in New York State. Environ. Health Perspect..

[15] Cory-Slechta D.A., Merrill A., Sobolewski M. (2023). Air Pollution–Related Neurotoxicity Across the Life Span. Annu. Rev. Pharmacol..

[16] Rump K., Adamzik M. (2022). Epigenetic Mechanisms of Postoperative Cognitive Impairment Induced by Anesthesia and Neuroinflammation. Cells.

[17] Harman M.F., Martín M.G. (2019). Epigenetic mechanisms related to cognitive decline during aging. J. Neurosci. Res..

[18] Maity S., Farrell K., Navabpour S., Narayanan S.N., Jarome T.J. (2021). Epigenetic Mechanisms in Memory and Cognitive Decline Associated with Aging and Alzheimer’s Disease. Int. J. Mol. Sci..

[19] Millán-Zambrano G., Burton A., Bannister A.J., Schneider R. (2022). Histone post-translational modifications—Cause and consequence of genome function. Nat. Rev. Genet..

[20] Xu J., Zhang Q., Su Z., Liu Y., Yan T., Zhang Y., Wang T., Wei X., Chen Z., Hu G. (2022). Genetic damage and potential mechanism exploration under different air pollution patterns by multi-omics. Environ. Int..

[21] Younesian S., Yousefi A.-M., Momeny M., Ghaffari S.H., Bashash D. (2022). The DNA Methylation in Neurological Diseases. Cells.

[22] Alcalà-Vida R., Awada A., Boutillier A.-L., Merienne K. (2021). Epigenetic mechanisms underlying enhancer modulation of neuronal identity, neuronal activity and neurodegeneration. Neurobiol. Dis..

[23] Xylaki M., Atzler B., Outeiro T.F. (2019). Epigenetics of the Synapse in Neurodegeneration. Curr. Neurol. Neurosci..

[24] Prasher D., Greenway S.C., Singh R.B. (2020). The impact of epigenetics on cardiovascular disease. Biochem. Cell Biol..

[25] Giallongo S., Longhitano L., Denaro S., D’Aprile S., Torrisi F., La Spina E., Giallongo C., Mannino G., Lo Furno D., Zappalà A. (2022). The Role of Epigenetics in Neuroinflammatory-Driven Diseases. Int. J. Mol. Sci..

[26] Li K., Liang X., Xie X., Tian L., Yan J., Lin B., Liu H., Lai W., Liu X., Xi Z. (2023). Role of SHANK3 in concentrated ambient PM2. 5 exposure induced autism-like phenotype. Heliyon.

[27] Moutin E., Sakkaki S., Compan V., Bouquier N., Giona F., Areias J., Goyet E., Hemonnot-Girard A.-L., Seube V., Glasson B. (2021). Restoring glutamate receptosome dynamics at synapses rescues autism-like deficits in Shank3-deficient mice. Mol. Psychiatry.

[28] Wei H., Liang F., Meng G., Nie Z., Zhou R., Cheng W., Wu X., Feng Y., Wang Y. (2016). Redox/methylation mediated abnormal DNA methylation as regulators of ambient fine particulate matter-induced neurodevelopment related impairment in human neuronal cells. Sci. Rep..

[29] Tononi G., Cirelli C. (2019). Sleep and synaptic down-selection. Eur. J. Neurosci..

[30] Khlghatyan J., Evstratova A., Bozoyan L., Chamberland S., Chatterjee D., Marakhovskaia A., Soares Silva T., Toth K., Mongrain V., Beaulieu J.M. (2020). Fxr1 regulates sleep and synaptic homeostasis. EMBO J..

[31] Hepsomali P., Coxon C. (2022). Inflammation and diet: Focus on mental and cognitive health. Adv. Clin. Exp. Med..

[32] Chen R., Meng X., Zhao A., Wang C., Yang C., Li H., Cai J., Zhao Z., Kan H. (2016). DNA hypomethylation and its mediation in the effects of fine particulate air pollution on cardiovascular biomarkers: A randomized crossover trial. Environ. Int..

[33] Zorkina Y., Abramova O., Ushakova V., Andreyuk D., Andriushchenko N., Pavlov K., Savilov V., Soloveva K., Kurmishev M., Syunyakov T. (2023). Inflammatory biomarkers and lipid metabolism parameters in women with mild cognitive impairment and dementia. Women Health.

[34] Lecca D., Jung Y.J., Scerba M.T., Hwang I., Kim Y.K., Kim S., Modrow S., Tweedie D., Hsueh S.C., Liu D. (2022). Role of chronic neuroinflammation in neuroplasticity and cognitive function: A hypothesis. Alzheimer’s Dement..

[35] Wang C., O’Brien K.M., Xu Z., Sandler D.P., Taylor J.A., Weinberg C.R. (2019). Long-term ambient fine particulate matter and DNA methylation in inflammation pathways: Results from the Sister Study. Epigenetics.

[36] Li Z., Liang D., Ebelt S., Gearing M., Kobor M.S., Konwar C., Maclsaac J.L., Dever K., Wingo A., Levey A. (2024). Differential DNA Methylation in the Brain as Potential Mediator of the Association between Traffic-related PM2.5 and Neuropathology Markers of Alzheimer’s Disease. Alzheimer’s Dement..

[37] Sordillo J.E., Cardenas A., Qi C., Rifas-Shiman S.L., Coull B., Luttmann-Gibson H., Schwartz J., Kloog I., Hivert M.-F., DeMeo D.L. (2021). Residential PM2.5 exposure and the nasal methylome in children. Environ. Int..

[38] Hasegawa-Ishii S., Shimada A., Imamura F. (2019). Neuroplastic changes in the olfactory bulb associated with nasal inflammation in mice. J. Allergy Clin. Immunol..

[39] Hasegawa Y., Namkung H., Smith A., Sakamoto S., Zhu X., Ishizuka K., Lane A.P., Sawa A., Kamiya A. (2021). Causal impact of local inflammation in the nasal cavity on higher brain function and cognition. Neurosci. Res..

[40] Yang Y., Yang T., Zhou J., Cao Z., Liao Z., Zhao Y., Su X., He J., Hua J. (2022). Prenatal exposure to concentrated ambient PM2.5 results in spatial memory defects regulated by DNA methylation in male mice offspring. Environ. Sci. Pollut. Res..

[41] Shou Y., Huang Y., Zhu X., Liu C., Hu Y., Wang H. (2019). A review of the possible associations between ambient PM2.5 exposures and the development of Alzheimer’s disease. Ecotoxicol. Environ. Saf..

[42] Louis S., Carlson A.K., Suresh A., Rim J., Mays M., Ontaneda D., Dhawan A. (2023). Impacts of Climate Change and Air Pollution on Neurologic Health, Disease, and Practice. Neurology.

[43] Kwon H.S., Koh S.-H. (2020). Neuroinflammation in neurodegenerative disorders: The roles of microglia and astrocytes. Transl. Neurodegener..

[44] Costa L.G., Cole T.B., Dao K., Chang Y.-C., Coburn J., Garrick J.M. (2020). Effects of air pollution on the nervous system and its possible role in neurodevelopmental and neurodegenerative disorders. Pharmacol. Therapeut.

[45] Saleh A., Macia A., Muotri A.R. (2019). Transposable Elements, Inflammation, and Neurological Disease. Front. Neurol..

[46] Hashimoto K., Tangsuwansri C., Saeliw T., Thongkorn S., Chonchaiya W., Suphapeetiporn K., Mutirangura A., Tencomnao T., Hu V.W., Sarachana T. (2018). Investigation of epigenetic regulatory networks associated with autism spectrum disorder (ASD) by integrated global LINE-1 methylation and gene expression profiling analyses. PLoS ONE.

[47] Zhou Y., Zhang M., Liu W., Li Y., Qin Y., Xu Y. (2020). Transgenerational transmission of neurodevelopmental disorders induced by maternal exposure to PM2.5. Chemosphere.

[48] Gao L., Zhang Y., Sterling K., Song W. (2022). Brain-derived neurotrophic factor in Alzheimer’s disease and its pharmaceutical potential. Transl. Neurodegener..

[49] Weaver J.R., Susiarjo M., Bartolomei M.S. (2009). Imprinting and epigenetic changes in the early embryo. Mamm. Genome.

[50] Johnson N.M., Hoffmann A.R., Behlen J.C., Lau C., Pendleton D., Harvey N., Shore R., Li Y., Chen J., Tian Y. (2021). Air pollution and children’s health—A review of adverse effects associated with prenatal exposure from fine to ultrafine particulate matter. Environ. Health Prev. Med..

[51] Tao S., Zhang X., Tian F., Pan B., Peng R., Wang Y., Xia M., Yang M., Hu J., Kan H. (2022). Maternal exposure to ambient PM2.5 causes fetal growth restriction via the inhibition of spiral artery remodeling in mice. Ecotoxicol. Environ. Saf..

[52] Yue H., Ji X., Li G., Hu M., Sang N. (2019). Maternal Exposure to PM2.5 Affects Fetal Lung Development at Sensitive Windows. Environ. Sci. Technol..

[53] Liu Y., Wang L., Wang F., Li C. (2016). Effect of Fine Particulate Matter (PM2.5) on Rat Placenta Pathology and Perinatal Outcomes. Med. Sci. Monit..

[54] Zhao Y., Wang P., Zhou Y., Xia B., Zhu Q., Ge W., Li J., Shi H., Xiao X., Zhang Y. (2021). Prenatal fine particulate matter exposure, placental DNA methylation changes, and fetal growth. Environ. Int..

[55] Xu C., Tao X., Ma X., Zhao R., Cao Z., Morishita R. (2021). Cognitive Dysfunction after Heart Disease: A Manifestation of the Heart-Brain Axis. Oxidative Med. Cell. Longev..

[56] Iadecola C., Gottesman R.F. (2019). Neurovascular and Cognitive Dysfunction in Hypertension. Circ. Res..

[57] Wang C., Chen R., Cai J., Shi J., Yang C., Tse L.A., Li H., Lin Z., Meng X., Liu C. (2016). Personal exposure to fine particulate matter and blood pressure: A role of angiotensin converting enzyme and its DNA methylation. Environ. Int..

[58] van Nieuwkerk A.C., Delewi R., Wolters F.J., Muller M., Daemen M., Biessels G.J. (2023). Cognitive Impairment in Patients With Cardiac Disease: Implications for Clinical Practice. Stroke.

[59] Faulkner K.M., Dickson V.V., Fletcher J., Katz S.D., Chang P.P., Gottesman R.F., Witt L.S., Shah A.M., D’Eramo Melkus G. (2022). Factors Associated With Cognitive Impairment in Heart Failure With Preserved Ejection Fraction. J. Cardiovasc. Nurs..

[60] Yang X., Feng L., Zhang Y., Hu H., Shi Y., Liang S., Zhao T., Fu Y., Duan J., Sun Z. (2018). Cytotoxicity induced by fine particulate matter (PM2.5) via mitochondria-mediated apoptosis pathway in human cardiomyocytes. Ecotoxicol. Environ. Saf..

[61] Yang X., Zhao T., Feng L., Shi Y., Jiang J., Liang S., Sun B., Xu Q., Duan J., Sun Z. (2019). PM2.5-induced ADRB2 hypermethylation contributed to cardiac dysfunction through cardiomyocytes apoptosis via PI3K/Akt pathway. Environ. Int..

[62] Grande G., Ljungman P.L.S., Eneroth K., Bellander T., Rizzuto D. (2020). Association Between Cardiovascular Disease and Long-term Exposure to Air Pollution With the Risk of Dementia. JAMA Neurol..

[63] Collins B.E., Greer C.B., Coleman B.C., Sweatt J.D. (2019). Histone H3 lysine K4 methylation and its role in learning and memory. Epigenetics & Chromatin.

[64] Zhang Y., Sun Z., Jia J., Du T., Zhang N., Tang Y., Fang Y., Fang D. (2021). Overview of Histone Modification. Histone Mutations and Cancer.

[65] Vrijens K., Trippas A.-J., Lefebvre W., Vanpoucke C., Penders J., Janssen B.G., Nawrot T.S. (2020). Association of Prenatal Exposure to Ambient Air Pollution With Circulating Histone Levels in Maternal Cord Blood. JAMA Netw. Open.

[66] Abrams S.T., Zhang N., Manson J., Liu T., Dart C., Baluwa F., Wang S.S., Brohi K., Kipar A., Yu W. (2013). Circulating Histones Are Mediators of Trauma-associated Lung Injury. Am. J. Respir. Crit. Care Med..

[67] Kresovich J.K., Zhang Z., Fang F., Zheng Y., Sanchez-Guerra M., Joyce B.T., Zhong J., Chervona Y., Wang S., Chang D. (2017). Histone 3 modifications and blood pressure in the Beijing Truck Driver Air Pollution Study. Biomarkers.

[68] Ungvari Z., Toth P., Tarantini S., Prodan C.I., Sorond F., Merkely B., Csiszar A. (2021). Hypertension-induced cognitive impairment: From pathophysiology to public health. Nat. Rev. Nephrol..

[69] Nativio R., Lan Y., Donahue G., Sidoli S., Berson A., Srinivasan A.R., Shcherbakova O., Amlie-Wolf A., Nie J., Cui X. (2020). An integrated multi-omics approach identifies epigenetic alterations associated with Alzheimer’s disease. Nat. Genet..

[70] Thiankhaw K., Chattipakorn N., Chattipakorn S.C. (2022). PM2.5 exposure in association with AD-related neuropathology and cognitive outcomes. Environ. Pollut..

[71] Ding R., Jin Y., Liu X., Zhu Z., Zhang Y., Wang T., Xu Y. (2016). H3K9 acetylation change patterns in rats after exposure to traffic-related air pollution. Environ. Toxicol. Pharmacol..

[72] Liu C., Xu J., Chen Y., Guo X., Zheng Y., Wang Q., Chen Y., Ni Y., Zhu Y., Joyce B.T. (2015). Characterization of genome-wide H3K27ac profiles reveals a distinct PM2.5-associated histone modification signature. Environ. Health.

[73] Li R., Zhao Y., Shi J., Zhao C., Xie P., Huang W., Yong T., Cai Z. (2020). Effects of PM2.5 exposure in utero on heart injury, histone acetylation and GATA4 expression in offspring mice. Chemosphere.

[74] Afouda B.A. (2022). Towards Understanding the Gene-Specific Roles of GATA Factors in Heart Development: Does GATA4 Lead the Way?. Int. J. Mol. Sci..

[75] Chen Z., Wu H., Zhang M. (2021). Long non-coding RNA: An underlying bridge linking neuroinflammation and central nervous system diseases. Neurochem. Int..

[76] Koh H., Lee S., Lee H., Min J.-W., Iwatsubo T., Teunissen C., Cho H.-J., Ryu J.-H. (2021). Targeting MicroRNA-485-3p Blocks Alzheimer’s Disease Progression. Int. J. Mol. Sci..

[77] Balusu S., Horré K., Thrupp N., Craessaerts K., Snellinx A., Serneels L., T’Syen D., Chrysidou I., Arranz A.M., Sierksma A. (2023). MEG3 activates necroptosis in human neuron xenografts modeling Alzheimer’s disease. Science.

[78] Yin Z., Herron S., Silveira S., Kleemann K., Gauthier C., Mallah D., Cheng Y., Margeta M.A., Pitts K.M., Barry J.-L. (2023). Identification of a protective microglial state mediated by miR-155 and interferon-γ signaling in a mouse model of Alzheimer’s disease. Nat. Neurosci..

[79] Yang Y.-S., He S.-L., Chen W.-C., Wang C.-M., Huang Q.-M., Shi Y.-C., Lin S., He H.-f. (2022). Recent progress on the role of non-coding RNA in postoperative cognitive dysfunction. Front. Cell. Neurosci..

[80] Soutschek M., Schratt G. (2023). Non-coding RNA in the wiring and remodeling of neural circuits. Neuron.

[81] Olufunmilayo E.O., Holsinger R.M.D. (2023). Roles of Non-Coding RNA in Alzheimer’s Disease Pathophysiology. Int. J. Mol. Sci..

[82] Aghaei-Zarch S.M., Alipourfard I., Rasoulzadeh H., Najafi S., Aghaei-Zarch F., Partov S., Movafagh A., Jahanara A., Toolabi A., Sheikhmohammadi A. (2023). Non-coding RNAs: An emerging player in particulate matter 2.5-mediated toxicity. Int. J. Biol. Macromol..

[83] Li Q., Li S., Xu C., Zhao J., Hou L., Jiang F., Zhu Z., Wang Y., Tian L. (2021). microRNA-149-5p mediates the PM2.5-induced inflammatory response by targeting TAB2 via MAPK and NF-κB signaling pathways in vivo and in vitro. Cell Biol. Toxicol..

[84] Vassiliou A., Vitsas V., Kardara M., Keskinidou C., Michalopoulou P., Rovina N., Dimopoulou I., Orfanos S.E., Tsoukalas G., Koutsoukou A. (2020). Study of Inflammatory Biomarkers in COPD and Asthma Exacerbations. Adv. Respir. Med..

[85] Muñoz-Delgado L., Labrador-Espinosa M.Á., Macías-García D., Jesús S., Benítez Zamora B., Fernández-Rodríguez P., Adarmes-Gómez A.D., Reina Castillo M.I., Castro-Labrador S., Silva-Rodríguez J. (2023). Peripheral Inflammation Is Associated with Dopaminergic Degeneration in Parkinson’s Disease. Mov. Disord..

[86] Costas C., Faro L.R.F. (2022). Do Naturally Occurring Antioxidants Protect Against Neurodegeneration of the Dopaminergic System? A Systematic Revision in Animal Models of Parkinson’s Disease. Curr. Neuropharmacol..

[87] Liu G., Li Y., Zhou J., Xu J., Yang B. (2022). PM2.5 deregulated microRNA and inflammatory microenvironment in lung injury. Environ. Toxicol. Pharmacol..

[88] Hu W., Wong J.Y.Y., Dai Y., Ren D., Blechter B., Duan H., Niu Y., Xu J., Fu W., Meliefste K. (2023). Occupational exposure to diesel engine exhaust and serum levels of microRNAs in a cross-sectional molecular epidemiology study in China. Environ. Mol. Mutagen..

[89] Feng L., Wei J., Liang S., Sun Z., Duan J. (2020). miR-205/IRAK2 signaling pathway is associated with urban airborne PM2.5-induced myocardial toxicity. Nanotoxicology.

[90] Li X., Jia Y., Nan A., Zhang N., Zhou H., Chen L., Pan X., Qiu M., Zhu J., Zhang H. (2020). CircRNA104250 and lncRNAuc001.dgp.1 promote the PM2.5-induced inflammatory response by co-targeting miR-3607-5p in BEAS-2B cells. Environ. Pollut..

[91] Emami M.H., Sereshki N., Malakoutikhah Z., Dehkordi S.A.E., Fahim A., Mohammadzadeh S., Maghool F. (2022). Nrf2 signaling pathway in trace metal carcinogenesis: A cross-talk between oxidative stress and angiogenesis. Comp. Biochem. Physiol. Part C Toxicol. Pharmacol..

[92] Yue W., Tong L., Liu X., Weng X., Chen X., Wang D., Dudley S.C., Weir E.K., Ding W., Lu Z. (2019). Short term Pm2.5 exposure caused a robust lung inflammation, vascular remodeling, and exacerbated transition from left ventricular failure to right ventricular hypertrophy. Redox Biol..

[93] Xu J., Xu H., Ma K., Wang Y., Niu B., Zhang L., Li F. (2021). lncRNA Gm16410 Mediates PM2.5-Induced Macrophage Activation via PI3K/AKT Pathway. Front. Cell Dev. Biol..

[94] Liao F., Tan Y., Wang Y., Zhou C., Wang Q., Li J., He L., Peng X. (2021). lncRNA AABR07005593.1 potentiates PM2.5-induced interleukin-6 expression by targeting MCCC1. Ecotoxicol. Environ. Saf..

[95] Xie J., Li S., Ma X., Li R., Zhang H., Li J., Yan X. (2022). MiR-217-5p inhibits smog (PM2.5)-induced inflammation and oxidative stress response of mouse lung tissues and macrophages through targeting STAT1. Aging.

[96] Peng Y.-H., Wu B.-R., Su C.-H., Liao W.-C., Muo C.-H., Hsia T.-C., Kao C.-H. (2015). Adult asthma increases dementia risk: A nationwide cohort study. J. Epidemiol. Community Health.

[97] Firoozi Z., Shahi A., Mohammadisoleimani E., Afzali S., Mansoori B., Bahmanyar M., Mohaghegh P., Dastsooz H., Pezeshki B., Nikfar G. (2024). CircRNA-associated ceRNA networks (circCeNETs) in chronic obstructive pulmonary disease (COPD). Life Sciences.

[98] Huang J., Hu Y., Wang Y., Jin Z. (2024). Activation of Notch1-GATA3 pathway in asthma bronchial epithelial cells induced by acute PM2.5 exposure and the potential protective role of microRNA-139-5p. J. Asthma.

[99] Wang L., Xu J., Liu H., Li J., Hao H. (2019). PM2.5 inhibits SOD1 expression by up-regulating microRNA-206 and promotes ROS accumulation and disease progression in asthmatic mice. Int. Immunopharm..

[100] Bairamian D., Sha S., Rolhion N., Sokol H., Dorothée G., Lemere C.A., Krantic S. (2022). Microbiota in neuroinflammation and synaptic dysfunction: A focus on Alzheimer’s disease. Mol. Neurodegener..

[101] Liu J., Liu B., Yuan P., Cheng L., Sun H., Gui J., Pan Y., Huang D., Chen H., Jiang L. (2021). Role of PKA/CREB/BDNF signaling in PM2.5-induced neurodevelopmental damage to the hippocampal neurons of rats. Ecotoxicol. Environ. Saf..

[102] Liu F., Liu C., Liu Y., Wang J., Wang Y., Yan B. (2023). Neurotoxicity of the air-borne particles: From molecular events to human diseases. J. Hazard. Mater..

[103] Li J., Wang Y., Steenland K., Liu P., van Donkelaar A., Martin R.V., Chang H.H., Caudle W.M., Schwartz J., Koutrakis P. (2022). Long-term effects of PM2.5 components on incident dementia in the northeastern United States. Innovation.

[104] Liu X.-q., Huang J., Song C., Zhang T.-l., Liu Y.-p., Yu L. (2023). Neurodevelopmental toxicity induced by PM2.5 Exposure and its possible role in Neurodegenerative and mental disorders. Hum. Exp. Toxicol..

[105] Chopra N., Wang R., Maloney B., Nho K., Beck J.S., Pourshafie N., Niculescu A., Saykin A.J., Rinaldi C., Counts S.E. (2020). MicroRNA-298 reduces levels of human amyloid-β precursor protein (APP), β-site APP-converting enzyme 1 (BACE1) and specific tau protein moieties. Mol. Psychiatry.

[106] Ku T., Li B., Gao R., Zhang Y., Yan W., Ji X., Li G., Sang N. (2017). NF-κB-regulated microRNA-574-5p underlies synaptic and cognitive impairment in response to atmospheric PM2.5 aspiration. Part. Fibre Toxicol..

[107] Yang J., Jia L., Li Y., Qiu Q., Quan M., Jia J. (2021). Fluid Biomarkers in Clinical Trials for Alzheimer’s Disease: Current and Future Application. J. Alzheimers Dis..

[108] Watson C.M., Dammer E.B., Ping L., Duong D.M., Modeste E., Carter E.K., Johnson E.C.B., Levey A.I., Lah J.J., Roberts B.R. (2023). Quantitative Mass Spectrometry Analysis of Cerebrospinal Fluid Protein Biomarkers in Alzheimer’s Disease. Sci. Data.

[109] Chauhan A., Chauhan V. (2020). Beneficial Effects of Walnuts on Cognition and Brain Health. Nutrients.

[110] Imbimbo B.P., Ippati S., Watling M., Imbimbo C. (2023). Role of monomeric amyloid-β in cognitive performance in Alzheimer’s disease: Insights from clinical trials with secretase inhibitors and monoclonal antibodies. Pharmacol. Res..

[111] Hou T., Liao J., Zhang C., Sun C., Li X., Wang G. (2018). Elevated expression of miR-146, miR-139 and miR-340 involved in regulating Th1/Th2 balance with acute exposure of fine particulate matter in mice. Int. Immunopharm..

[112] Tan X., Luo Y., Pi D., Xia L., Li Z., Tu Q. (2020). MiR-340 Reduces the Accumulation of Amyloid-β Through Targeting BACE1 (β-site Amyloid Precursor Protein Cleaving Enzyme 1) in Alzheimer’s Disease. Curr. Neurovasc. Res..

[113] Liu C.-g., Wang J.-l., Li L., Xue L.-x., Zhang Y.-q., Wang P.-c. (2014). MicroRNA-135a and -200b, potential Biomarkers for Alzheimer׳s disease, regulate β secretase and amyloid precursor protein. Brain Res..

[114] Lee H.-W. (2017). Elevated microRNA-135a is associated with pulmonary arterial hypertension in experimental mouse model. Oncotarget.

[115] Wegmann S., Biernat J., Mandelkow E. (2021). A current view on Tau protein phosphorylation in Alzheimer’s disease. Curr. Opin. Neurobiol..

[116] Henneghan A., Haley A.P., Kesler S. (2019). Exploring Relationships Among Peripheral Amyloid Beta, Tau, Cytokines, Cognitive Function, and Psychosomatic Symptoms in Breast Cancer Survivors. Biol. Res. Nurs..

[117] Opland C.K., Bryan M.R., Harris B., McGillion-Moore J., Tian X., Chen Y., Itano M.S., Diering G.H., Meeker R.B., Cohen T.J. (2023). Activity-dependent tau cleavage by caspase-3 promotes neuronal dysfunction and synaptotoxicity. iScience.

[118] Zhang Y., Zhang Y., Aman Y., Ng C.T., Chau W.-H., Zhang Z., Yue M., Bohm C., Jia Y., Li S. (2021). Amyloid-β toxicity modulates tau phosphorylation through the PAX6 signalling pathway. Brain.

[119] Ionescu-Tucker A., Cotman C.W. (2021). Emerging roles of oxidative stress in brain aging and Alzheimer’s disease. Neurobiol. Aging.

[120] Feng S., Gao D., Liao F., Zhou F., Wang X. (2016). The health effects of ambient PM2.5 and potential mechanisms. Ecotoxicol. Environ. Saf..

[121] Moufarrej L., Verdin A., Cazier F., Ledoux F., Courcot D. (2023). Oxidative stress response in pulmonary cells exposed to different fractions of PM2.5-0.3 from urban, traffic and industrial sites. Environ. Res..

[122] Kumari S., Prakash S., Gupta S.K. (2021). NRF2: A key regulator of endothelial microRNA transcription. Cardiovasc. Res..

[123] Wang X., Zhu H., Sun G., Zhou M., Zhang H., Liu H., Wang M., Zhang Z., Chu H. (2023). linc01515 regulates PM2.5-induced oxidative stress via targeting NRF2 in airway epithelial cells. Environ. Pollut..

[124] Li B., Huang N., Wei S., Xv J., Meng Q., Aschner M., Li X., Chen R. (2021). lncRNA TUG1 as a ceRNA promotes PM exposure-induced airway hyper-reactivity. J. Hazard. Mater..

[125] Song J., Qu R., Sun B., Chen R., Kan H., An Z., Jiang J., Li J., Zhang Y., Wu W. (2021). Associations of Short-Term Exposure to Fine Particulate Matter with Neural Damage Biomarkers: A Panel Study of Healthy Retired Adults. Environ. Sci. Technol..

[126] Ikram M.F., Farhat S.M., Mahboob A., Baig S., Yaqinuddin A., Ahmed T. (2020). Expression of DnMTs and MBDs in AlCl3-Induced Neurotoxicity Mouse Model. Biol. Trace Elem. Res..

[127] Pan B., Zhou Y., Li H., Li Y., Xue X., Li L., Liu Q., Zhao X., Niu Q. (2020). Relationship between occupational aluminium exposure and histone lysine modification through methylation. J. Trace Elem. Med. Biol..

[128] Zhou C., Liu M., Mei X., Li Q., Zhang W., Deng P., He Z., Xi Y., Tong T., Pi H. (2021). Histone hypoacetylation contributes to neurotoxicity induced by chronic nickel exposure in vivo and in vitro. Sci. Total Environ..

[129] Searles Nielsen S., Checkoway H., Criswell S.R., Farin F.M., Stapleton P.L., Sheppard L., Racette B.A. (2015). Inducible nitric oxide synthase gene methylation and parkinsonism in manganese-exposed welders. Park. Relat. D.

[130] Olasehinde T.A., Olaniran A.O. (2022). Neurotoxicity of Polycyclic Aromatic Hydrocarbons: A Systematic Mapping and Review of Neuropathological Mechanisms. Toxics.

[131] Li Y., Cao J., Hao Z., Liu A., Li X., Li H., Xia N., Wang Z., Zhang Z., Bai J. (2022). Aspirin ameliorates the cognition impairment in mice following benzo[a]pyrene treatment via down-regulating BDNF IV methylation. Neurotoxicology.

[132] Wang J., Li C.-L., Tu B.-J., Yang K., Mo T.-T., Zhang R.-Y., Cheng S.-Q., Chen C.-Z., Jiang X.-J., Han T.-L. (2018). Integrated epigenetics, transcriptomics, and metabolomics to analyze the mechanisms of Benzo[a]pyrene neurotoxicity in the hippocampus. Toxicol. Sci..

