# Supplementary material for: The Role of Epigenetic Mechanisms in the Development of PM2.5-Induced Cognitive Impairment
Source: Toxics. 2025 Feb 2;13(2):119. doi: 10.3390/toxics13020119 (PMC11861554; doi:10.3390/toxics13020119)
Supplement: Supplementary file 1 [file toxics-13-00119-s001.zip › toxics-3402567-supplementary.pdf]

Supplementary Materials:

Table S1. The searching string.

Database	Searching String
PubMed	("PM2.5"[All Fields] OR ("air pollution"[MeSH Terms] OR ("air"[All Fields] AND "pollution"[All Fields]) OR "air pollution"[All Fields]) OR ("fine"[All Fields] AND ("particulate"[All Fields] OR "particulates"[All Fields]))) AND ((("PM2.5"[All Fields] OR ("air pollution"[MeSH Terms] OR ("air"[All Fields] AND "pollution"[All Fields]) OR "air pollution"[All Fields]) OR ("fine"[All Fields] AND ("particulate"[All Fields] OR "particulates"[All Fields]))) AND ("cognitive dysfunction"[MeSH Terms] OR ("cognitive"[All Fields] AND "dysfunction"[All Fields]) OR "cognitive dysfunction"[All Fields] OR ("cognitive"[All Fields] AND "disorder"[All Fields]) OR "cognitive disorder"[All Fields])) OR (("cognition"[MeSH Terms] OR "cognition"[All Fields] OR "cognitions"[All Fields] OR "cognitive"[All Fields] OR "cognitively"[All Fields] OR "cognitives"[All Fields]) AND "dysfuction"[All Fields]))<br><br>("PM2.5"[All Fields] OR ("fine"[All Fields] AND ("particulate"[All Fields] OR "particulates"[All Fields])) OR ("air pollution"[MeSH Terms] OR ("air"[All Fields] AND "pollution"[All Fields]) OR "air pollution"[All Fields])) AND ("epigenetical"[All Fields] OR "epigenetically"[All Fields] OR "epigenomics"[MeSH Terms] OR "epigenomics"[All Fields] OR "epigenetic"[All Fields] OR "epigenetics"[All Fields] OR ("dna methylation"[MeSH Terms] OR ("dna"[All Fields] AND "methylation"[All Fields]) OR "dna methylation"[All Fields]) OR (("histon"[All Fields] OR "histones"[MeSH Terms] OR "histones"[All Fields] OR "histone"[All Fields] OR "histonic"[All Fields] OR "histons"[All Fields]) AND ("modification"[All Fields] OR "modifications"[All Fields])) OR ("ncrnas"[All Fields] OR "rna, untranslated"[MeSH Terms] OR ("rna"[All Fields] AND "untranslated"[All Fields]) OR "untranslated rna"[All Fields] OR "ncrna"[All Fields]) OR ("rna, untranslated"[MeSH Terms] OR ("rna"[All Fields] AND "untranslated"[All Fields]) OR "untranslated rna"[All Fields] OR ("non"[All Fields] AND "coding"[All Fields] AND "rna"[All Fields]) OR "non coding rna"[All Fields]))

Figure S1. PRISMA flow chart on article searching.

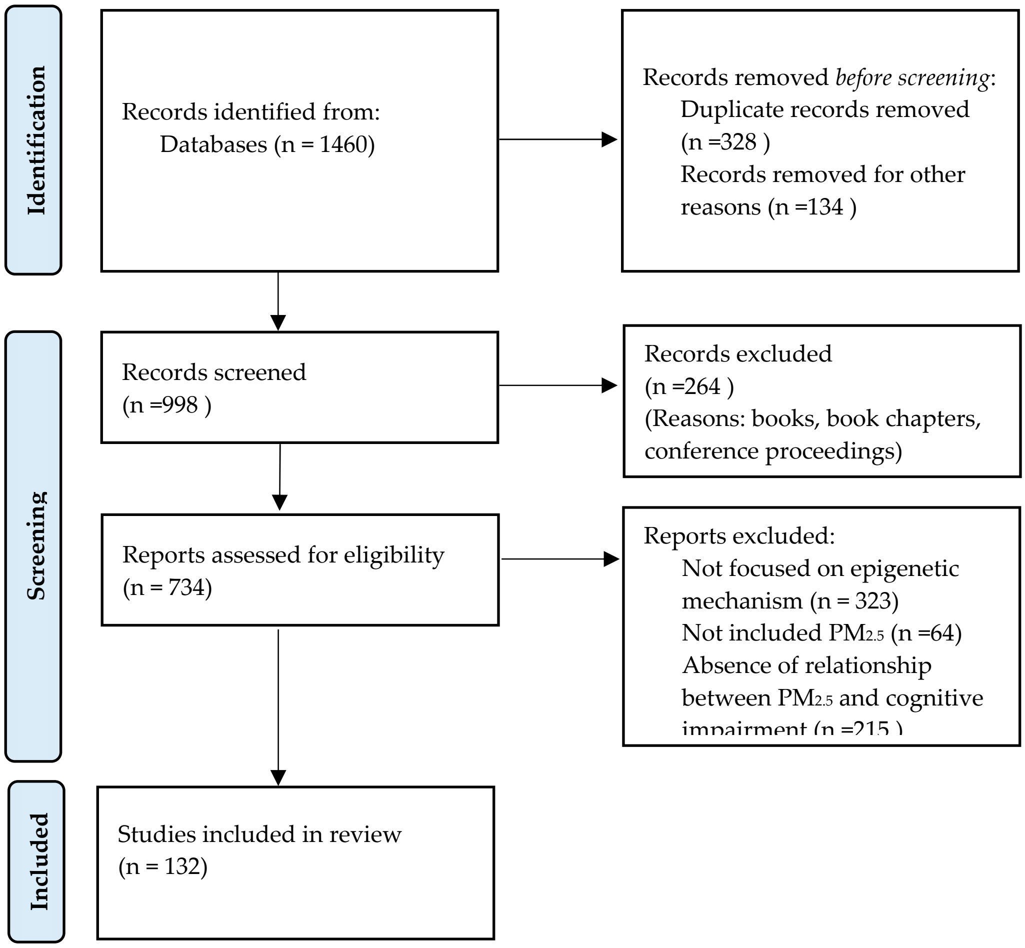

Table S2. PECO (population, exposure, comparator, and outcome) model.

Variable	Description		
Population	Human population studies, animal experiments, and neuron cells in vitro.		
	Human	Animal	In vitro cell
Exposure	Inhalation.	Inhalation.	Direct exposure to target cell.
Comparator	Population with low exposure risk or without exposure risk.<br>Self-control.	Animal without exposure.	Cells cultured without exposure.
Outcomes	Altered DNA methylation or histone modification or ncRNA.<br>Cognitive impairment.<br>Cardiac dysfunction.<br>Hypertension.	Altered DNA methylation or histone modification or ncRNA.<br>Increase in biomarkers and inflammatory factors.<br>Neurodevelopmental disorder.<br>Cardiac dysfunction.<br>Accumulation of ROS.	Altered DNA methylation or histone modification or ncRNA.<br>Apoptosis.

The criteria of selection and exclusion were determined for the review process according to population, exposure, comparator, and outcomes, or the PECO model. Only research articles containing empirical data were selected.
